# Reversal effect of FW-04-806, a macrolide dilactone compound, on multidrug resistance mediated by ABCB1 and ABCG2 in vitro and in vivo

**DOI:** 10.1186/s12964-019-0408-5

**Published:** 2019-09-01

**Authors:** Zhiqiang Zhang, Chunling Ma, Peng Li, Min Wu, Shengnan Ye, Liwu Fu, Jianhua Xu

**Affiliations:** 1 Department of Pharmacology, School of Pharmacy, Fujian Provincial Key Laboratory of Natural Medicine Pharmacology, Fujian Medical University, Fuzhou, 350122 China; 2 State Key Laboratory of Oncology in South China, Collaborative Innovation Center for Cancer Medicine, Guangdong Esophageal Cancer Institute, Sun Yat-sen University Cancer Center, Guangzhou, 510060 China; 3 The First Affiliated Hospital of Fujian Medical University, Fuzhou, 350004 China

**Keywords:** FW-04-806, ABCB1 transporter, ABCG2 transporter, Multiple drug resistance, Molecular docking

## Abstract

**Background:**

Overexpression of ATP-binding cassette (ABC) transporters, such as ABCB1 and ABCG2, has been proved to be a major trigger for multidrug resistance (MDR) in certain types of cancer. A promising approach to reverse MDR is the combined use of nontoxic and potent ABC transporters inhibitor with conventional anticancer drugs. We previously reported that FW-04-806 (conglobatin) as a novel Hsp90 inhibitor with low toxicity, capable of attenuating Hsp90/Cdc37 /clients interactions and producing antitumor action in vitro and in vivo. Our early activity screening found that FW-04-806 at non-cytotoxic concentration was able to enhance the cytotoxicity of chemotherapeutic agents on the ABCB1 overexpressing cells. Therefore, we speculated that FW-04-806 might be a promising MDR reversal agent. In the present study we further investigated its reversal effect of MDR induced by ABC transporters in vitro and in vivo.

**Methods:**

MTT assay in vitro and xenograftes in vivo were used to investigate reversal effect of FW-04-806 on MDR in ABCB1 or ABCG2 overexpressing cancer cells. To understand the mechanisms for the MDR reversal, we examined the effects of FW-04-806 on intracellular accumulation of doxorubicin (DOX, adriamycin, adr)/Rhodamine 123 (Rho 123), efflux of doxorubicin, expression levels of gene and protein of ABCB1 or ABCG2 and ATPase activity of ABCB1, and carried out molecular docking between FW-04-806 and human ABCB1.

**Results:**

The results indicated that FW-04-806 significantly enhanced the cytotoxicity of substrate chemotherapeutic agents on the ABCB1 or ABCG2 overexpressing cells in vitro and in vivo suggesting its reversal MDR effects. FW-04-806 increased the intracellular accumulation of DOX or Rho123 by inhibiting the efflux function of ABC transporters in MDR cells rather than in their parental sensitive cells. However, unlike other ABC transporter inhibitors, FW-04-806 had no effect on the ATPase activity nor on the expression of ABCB1 or ABCG2 on either mRNA or protein level. Molecular docking suggested that FW-04-806 may have lower affinity to the ATPase site, which was consistent with its no significant effect on the ATPase activity of ABCB1; However FW-04-806 may bind to substrate binding site in TMDs more stably than substrate anticancer drugs therefore obstruct the anticancer drugs pumped out of the cell.

**Conclusions:**

FW-04-806 is a compound that has both anti-tumor and reversal MDR effects, and its antitumor clinical application is worth further study.

**Graphical abstract:**

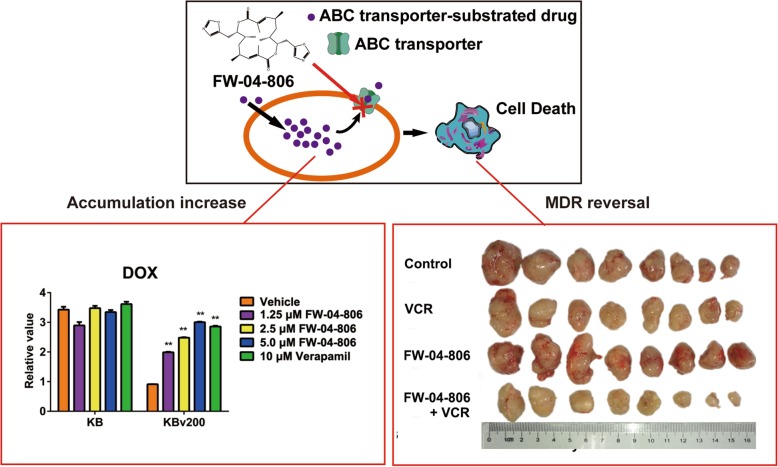

## Background

Chemotherapy is the main treatment for many malignant tumors. Multidrug resistance (MDR) in cancer cells produces cross-resistance towards a huge cluster of structurally and functionally unrelated chemotherapeutic drugs and this significantly decreases the efficacy of cancer chemotherapy [1]. The most common mechanism of MDR is the overexpression of ABC transporters, which actively pump numerous chemotherapeutic drugs out from cancer cells, thereby attenuating the efficacy of chemotherapeutic agents [2]. Currently, 49 members of the ABC transporter family have been identified and are divided into seven subfamilies (from ABCA to ABCG) based on the sequence and structure similarities [3, 4]. The subfamily ABCB1/MDR1/P-glycoprotein, ABCC1/MRP1, and ABCG2/BCRP play a major role in producing MDR in tumor cells [5]. ABCB1 (MDR1/P-gp) was the first eukaryotic ABC transporter identified that confers MDR in cancer cells [6]. ABCB1, is a 170 kDa membrane-associated glycoprotein, is composed of two homologous halves, each containing six transmembrane domains and an ATP binding domain, separated by a flexible polypeptide linker [7]. It can transport a wide range of antineoplastic drugs such as the anthracyclines, vinca alkaloids, taxanes, and epipodophyllotoxins [8]. ABCG2 is known as a half transporter that effluxes agents with amphiphilic characteristics [9, 10]. It can actively efflux a wide variety of antineoplastic drugs including mitoxantrone, topoisomerase I inhibitors and anthracyclines, as well as fluorescent dyes such as Hoechst 33342 [11]. ABCC1 is a 190-kDa protein that was first discovered in DOX resistant HL60/adr and H69AR cell lines [12, 13]. Its substrates include vinblastine (VLB), vincristine (VCR) and so on.

Since the discovery of ABC transporter inhibitor, combined use of the inhibitor with conventional anticancer drugs may represent a promising strategy to circumvent MDR [14]. To date, three generations of MDR inhibitors have been developed, some of which are currently under clinical trials to evaluate their usefulness in circumventing anticancer drug resistance [2]. However, many of these inhibitors failed to be utilized in clinical application due to the unacceptable toxicity as well as problematic pharmacokinetic interactions [15]. Screening of MDR reversers will provide new sources for research and development of new MDR reversal agents.

FW-04-806, a macrolide dilactone compound with double-oxazole ring, was isolated from the fermentation product of the China-native Streptomyces FIM-04-806, and was identified to be conglobatin [16, 17]. We previously reported that FW-04-806 was a Hsp90 inhibitor with low toxicity, and protein binding experiments and immunoprecipitation had shown that FW-04-806 could interfere with Hsp90 and Cdc37 binding, mediate the degradation of a series of Hsp90 client proteins and produce antitumor action in vitro and in vivo [18]. Based on the early activity screening, we found that FW-04-806 at a non-cytotoxic concentrations could enhance the cytotoxicity of chemotherapeutic agents on the ABCB1-overexpressing cells in vitro. Therefore, we speculate that FW-04-806 may be a promising MDR reversal agent. This work was intended to investgate FW-04-806 on reversing MDR mediated by ABCB1 or ABCG2 and its mechenism.

## Materials and methods

### Chemicals and reagents

Doxorubicin (DOX), paclitaxel, vincristine (VCR), cisplatin, topotecan, mitoxantrone (MX), verapamil (VRP), KO134, rhodamine123 (Rho123), 3-(4, 5-dimethylthiazol-2-yl)-2, 5-diphenyltetrazolium bromide (MTT) and dimethyl sulfoxide (DMSO) were all purchased from Sigma-Aldrich (St. Louis, MO, USA). SYBR Green qPCR Master Mix was purchased from TaKaRa. Dulbecco’s modified Eagle medium (DMEM) and RPMI-1640 were obtained from Gibco BRL (Thermo Fisher Scientific Inc., Waltham, MA, USA). Monoclonal antibodies against ABCB1, ABCG2, glyceraldehyd-3-phosphate dehydrogenase (GAPDH) were from Santa Cruz Biotechnology (Santa Cruz, CA, USA). FW-04-806 with a purity of more than 98% was obtained from Institute of Materia Medica, Fujian Medical University (Fuzhou, China).

### Cell lines

KB (human oral epidermoid carcinoma cell line) and its vincristine-selected ABCB1 overexpressing cell line KBv200 [19], K562 (human chronic myelogenous leukemia cell line) and its doxorubicin selected ABCB1 overexpressing cell line K562/adr were cultured in RPMI 1640 medium supplemented with 10% FBS in the presence of 5% CO_2_ at 37 °C. MCF-7 (human breast carcinoma cell line) and its doxorubicin selected ABCB1 overexpressing cell line MCF-7/adr [20], S1 (human colon carcinoma cell line) and its mitoxantrone-selected ABCG2-overexpressing cell line S1-M1-80 [21], H460 (human non-small cell lung cancer cell line) and its MX-selected ABCG2-overexpressing cell line H460/MX20, HEK293 (human embryonic kidney cell line) and its empty vector or ABCB1, ABCG2 expressing vector stable transfecting cell lines HEK293/pcDNA3.1, HEK293/ABCB1, HEK293/ABCG2-R2 [22] were cultured in DMEM medium supplemented with 10% FBS in the presence of 5% CO_2_ at 37 °C. The above commercial cell lines were originally obtained from American type culture collection (ATCC). HEK293 and its transfected cell lines were kind gift provided by Dr. Susan Bates (National Cancer Institute, NIH, Bethesda, MD, U.S.A). The transfected cells were cultured in DMEM medium containing 2 mg/mL G418. All cells were grown in drug-free culture medium for more than 2 weeks before assay.

### Cell cytotoxicity assay

MTT assay was used to tests cell cytotoxicity and a drug concentration required to inhibit cell growth by 50% (IC_50_) was used to measure the sensitivity of cell to chemotherapeutic agents as described previously [23]. Briefly, cells growing in logarithmic phase were seeded at a density of 2000~7000 cells per well in 96-well plates. When the cells became adherent 24 h later, a range of different concentrations of conventional chemotherapeutic drugs with or without a fixed combination of FW-04-806 were added to the wells. After 72 h incubation, MTT (5 mg/mL, 20 μL) was added into each well, and 4 h later, the medium was discarded and 150 μL DMSO was added into the wells to dissolve the formazan product from the metabolism of MTT. Finally, optical density was measured at 540 nm by a Model 550 Microplate Reader (Bio-Rad, Hercules, CA, USA). IC_50_ was calculated from survival curves using the Bliss method [24]. The fold of resistance MDR cells to a drug was calculated by dividing the IC_50_ of the drug for the MDR cells by that of the same drug for the parental sensitive cells. The reversal degree (fold reversal) of a reversal agent for MDR was calculated by dividing the IC_50_ of a chemotherapeutic agent to MDR cells without a reversal agent by that of the chemotherapeutic agent with the reversal agent. All experiments were repeated at least three times.

### Animal experiments

KBv200 cell xenograft model was established as described previously [25]. Briefly, KBv200 cells at the logarithmic growth phase (2 × 10^7^ /0.2 mL/mouse) were inoculated into axillary subcutaneous of athymic nude mice (BALB/c-nu, male, 5 to 6 weeks of age, 18 to 22 g of weight, purchased from Shanghai SLAC Laboratory Animal Co. LTD.). When the tumor volume reached to 5 × 5 mm^3^, the mice were randomized into four groups by tumor size and received various treatments: (a) vehicle (gavage/p.o., q3d); (b) vincristine (0.5 mg/kg, tail vein injection/i.v., q3d); (c) FW-04-806 (100 mg/kg, gavage/p.o., q3d); (d) FW-04-806 (100 mg/kg, gavage/p.o., q3d, given 1 h before injecting vincristine) plus vincristine (0.5 mg/kg, tail vein injection/i.v., q3d). The body weights of the animals, tumor’s long diameter (L) and short diameter (W) were recorded every 3 days, and tumor volume (V) was estimated according to the following formula: V = L × W^2^/2. At the end of the observation period, the mice were euthanized and the xenografts were excised and weighed. The curves of tumor growth was drawn according to tumor volume and days after treatment. The ratio of growth inhibition (IR) was calculated according to the following formula [25]:

$$ \mathrm{IR}\ \left(\%\right)=\left(1-\frac{\mathrm{Mean}\ \mathrm{tumor}\ \mathrm{weight}\ \mathrm{of}\ \mathrm{experimental}\ \mathrm{group}}{\mathrm{Mean}\ \mathrm{tumor}\ \mathrm{weight}\ \mathrm{of}\ \mathrm{control}\ \mathrm{group}}\right)\times 100 $$

The animal experiment protocols were approved by animal care and use committee, Fujian Medical University, China**.**

### DOX and Rho 123 accumulation assay

Flow cytometry assay was performed to measure the effects of FW-04-806 on the intracellular accumulation of DOX or Rho 123 in cancer cells as previously described [19]. Briefly, cells were seeded in six well plates overnight and were pretreated with different concentrations of FW-04-806 or vehicle at 37 °C for 3 h; DOX (10 μM) or Rho 123 (5 μg/mL) was added to the medium for further incubation for another 3 h or 0.5 h respectively, then the cells were collected, centrifuged, and washed 3 times with chilled phosphate buffered saline (PBS). Finally, the cells were resuspended in 500 μL PBS and analyzed by flow cytometry (Cytomics FC500, Beckman Coulter Inc., Brea, CA, USA). Verapamil, a known ABCB1 inhibitor, was used as a positive control for ABCB1- overexpressing cells [26]. KO134, a specific ABCG2 inhibitor, was used as a positive control for ABCG2-overexpressing cells [27].

### DOX efflux assay

DOX efflux was performed as described earlier [28]. KBv200 or S1-MI-80 cells were pre-treated with 10 μM DOX at 37 °C for 3 h. The cells were washed three times with PBS and incubated with fresh culture media in the presence or absence of 5 μM FW-04-806 for KBv200 cells or 10 μM FW-04-806 for S1-MI-80 cells at 37 °C for 0, 15, 30, 60 and 90 min respectively. Then, the cells were collected at the different time points and washed three times with ice-cold PBS. Finally, the cells were resuspended in ice-cold PBS buffer for flow cytometric analysis immediately.

### ABCB1 ATPase activity assay

ABCB1 ATPase Activity Assay Kit (Promega, V3601) was used to evaluate the effect of FW-04-806 on ATPase activity of recombinant human ABCB1. We prepared a series of concentrations of FW-04-806 solution to be tested according to the manufacturer’s protocol.

### Western blot analysis

Western blot analysis was performed as previously described [29]. The cells were treated with a range of different concentrations of FW-04-806 and were collected at time points. The proteins were extracted and the concentrations were determined using the BCA Protein Assay kit (Thermo Scientific). Equal amounts of proteins were separated by SDS-PAGE and transferred to polyvinylidene difluoride membranes. After blocking with 5% non-fat milk for 2 h at room temperature, the membranes were incubated with the indicated primary antibodies overnight at 4 °C. The membranes were then washed three times and incubated with horseradish peroxidase conjugated secondary antibody for 2 h at room temperature. After three washes, proteins were detected using the chemiluminescent detection reagents. GAPDH was used as a loading control.

### Real-time quantitative PCR

ABCB1 and ABCG2 mRNA expression level was assayed as previously described [30]. After treatment of FW-04-806, total cellular RNA was isolated by Trizol Reagent RNA extraction kit. The first strand cDNA was synthesized by cDNA reverse transcription kit (TaKaRa). Real-time quantitative PCR primers were 5′-CAGGCTTGCTGTAATTACCCA-3′(forward) and 5′-TCAAAGAAACAACGGTTCGG-3′(reverse) for ABCB1, 5′-TGGCTGTCATGGCTTCAGTA-3′(forward) and 5′-GCCACGTGATTCTTCCACAA-3′(reverse) for ABCG2, 5′-GAGTCAAGGATTTGGTCGT-3′(forward) and 5′-GATCTCGCTCCTGGAAGATG-3′(reverse) for GAPDH, respectively. SYBR Green Assay kit (TaKaRa) was used for real time PCR reaction, following manufacturer’s protocol. Relative quantification of ABCB1 or ABCG2 was analyzed using the 2^-^^△△Ct^ method as a ratio relative to the GAPDH expression level in each sample, respectively.

### Docking analysis

To understand the possible interaction between FW-04-806 and ABCB1, molecular docking study was carried out. We used the molecular structure of human P-glycoprotein (PDB ID: 6C0V) provided by Y. Kim, which contains two cytoplasmic nucleotide binding domains (NBDs) and two transmembrane domains (TMDs) [31]. The two NBDs together bind and hydrolyse ATP to provide a driving force for transport, while the TMDs are involved in substrate recognition and translocation across the lipid membranes (Fig. 7a–b) [32]. Before performing ligand docking, Multi-Channel Surface Mode was adopted to search for the binding pocket and to generate the protomol of the P-glycoprotein [33]. Then the energy minimized structure of FW-04-806, Doxorubicin (DOX) and Paclitaxel (PTX) were subjected to Surflex-Dock program in SYBYL-X 2.0 version. The docking scores are expressed in units of -lgK_d_ to evaluate the docking results, where K_d_ represents a dissociation constant of a ligand; The scores represent the affinity between the ligand and the P-glycoprotein [34].

**Fig. 1:**
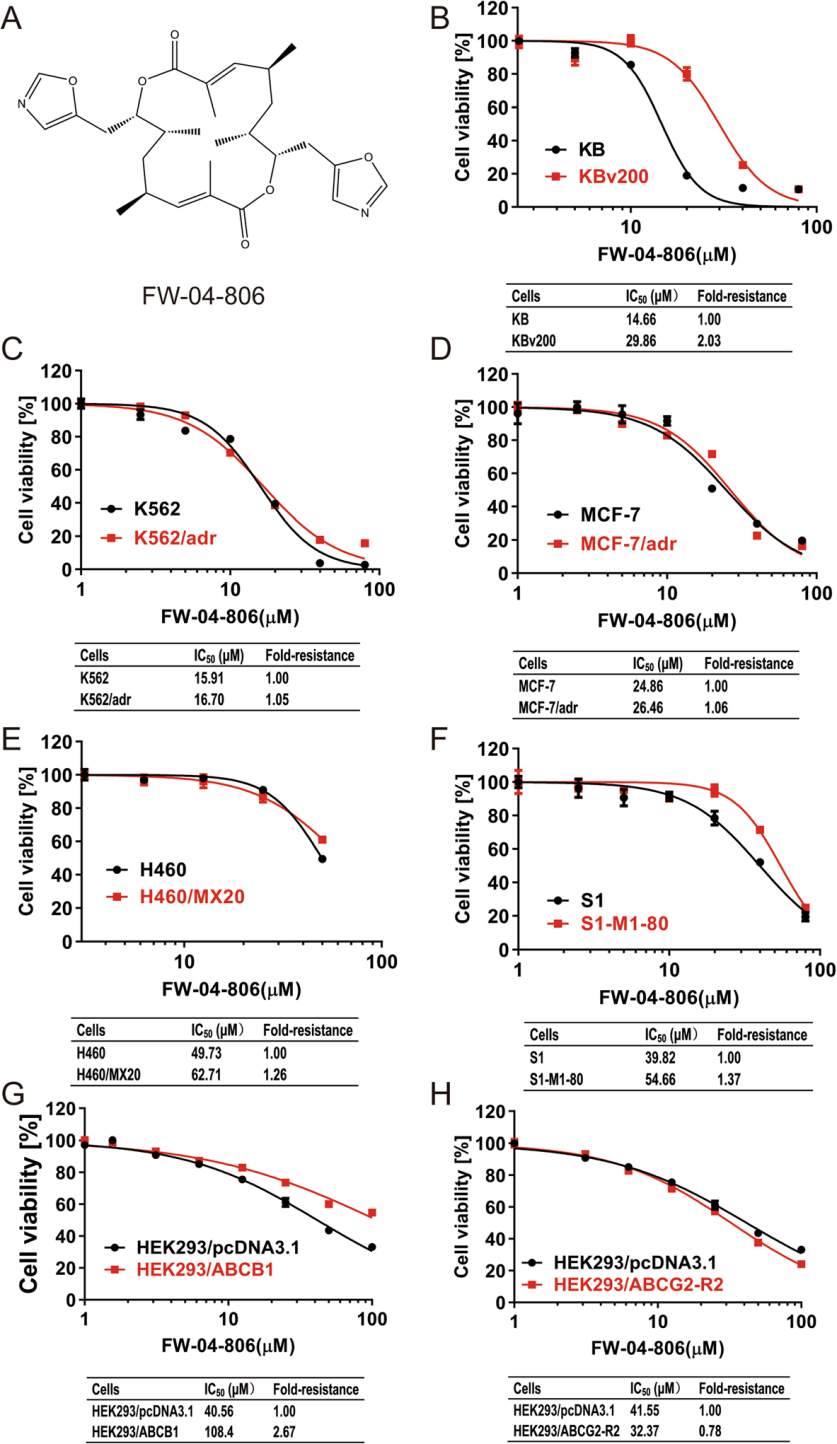
The structure of FW-04-806 and its cytotoxicity in drug resistant cell lines and their parental cell lines. The structure of FW-04-806 (**a**). Cytotoxicity of FW-04-806 on KBv200 and KB cells (**b**), S1-MI-80 and S1cells (**c**), K562/adr and K562 cells (**d**), H460/MX20 and H460 cells (**e**), MCF-7/adr and MCF-7 cells (**f**), HEK293/pcDNA3.1 and stable ABCG2 transfected HEK293/ABCG2-R2 cells (**g**) and HEK293/pcDNA3.1 and stable ABCB1 transfected HEK293/ABCB1 cells (**h**). All cells were treated with different concentrations of FW-04-806 for 72 h. Data are expressed as mean ± SD from three independent experiments

### Statistical analysis

Results were shown as mean ± SD. All experiments were repeated at least three times, and the differences were determined by using the Student *t* test. The statistical significance was determined to be “^*^” *P* < 0.05 and “^**^” *P* < 0.01.

## Results

### FW-04-806 enhanced the efficacy of substrate chemotherapeutic agents in ABCB1 and ABCG2 overexpressing cells in vitro

The structure of FW-04-806 is shown in Fig. 1a. To find out the suitable concentration of FW-04-806 for reversing MDR in vitro, we firstly examined the cytotoxic effect of FW-04-806 on different cell lines by MTT assay. As shown in Fig. 1b~h, the results showed that the IC_50_ values of FW-04-806 in ABCB1 overexpressing cell lines were all more than 13 μM and that in ABCG2 overexpressing cell lines all more than 50 μM. Thus, we chose concentrations of 5 μmol/ L and 10 μmol/L FW-04-806 as the maximum concentration for further reversal assay in ABCB1 and ABCG2 overexpressing cell lines respectively, at the chosen concentrations more than 80% of cells surviving. In addition, the IC_50_ values of FW-04-806 in ABCB1 or ABCG2 overexpressing cell lines were much closer to that in their parental sensitive cells, and the range of fold-resistance in MDR cell lines was from 0.78 to 2.67 (Fig. 1b~h), which similar to that of non-substrate drugs in the same MDR cell lines (from 0.5 to 5.8) (Table 2). These data suggested that FW-04-806 may be a non-substrate drug of ABC transporters. Then we investigated reversal effect of FW-04-806 on MDR in ABCB1 or ABCG2 overexpressing cancer cells. As expected, the IC_50_ of substrate chemotherapeutic agents such as doxorubicin, vincristine, paclitaxel, mitoxantrone, topotecan in ABCB1or ABCG2 overexpressing cell lines were higher than in the corresponding parental sensitive cell lines, and the range of fold resistance was from 20.9 to 1705.5 which suggested that ABCB1 or ABCG2 overexpressing cells were extremely resistant to substrate chemotherapeutic agents compared with their parental sensitive cells (Table 1). However, treatment with FW-04-806 significantly decreased the IC_50_ of the substrate chemotherapeutic agents in resistant cell lines overexpressing ABCB1 or ABCG2, showed that FW-04-806 was able to enhance the effects of the chemotherapeutic agents for MDR cells, which suggested its effects on reversal MDR, and the reversal effect was concentration dependence of FW-04-806. The range of the highest reversal fold in different MDR cell lines were from 7.28 to 308.55 and from 2.54 to 4.75 for overexpressing ABCB1 and ABCG2 cells repectively; While the IC_50_ of the substrate chemotherapeutic agents showed no obvious change in the corresponding parental sensitive cells treated with FW-04-806 (Table 1). Meanwhile, FW-04-806 did not decrease the IC_50_ of cisplatin, which is not a substrate of ABCB1 or ABCG2, in the ABCB1 or ABCG2 overexpressing MDR cells (Table 2). Since chemotherapeutic MDR results from various mechanisms, in order to demonstrate that FW-04-806 enhanced the efficacy of substrate chemotherapeutic agents in MDR cells was limited to ABCB1- and ABCG2-overexpressing cells, ABCB1 transfected HEK293/ABCB1 cells or ABCG2-R2 transfected HEK293/ABCG2-R2 cells were used to study the reversal MDR of FW-04-806. As anticipated, FW-04-806 had reversal effects of ABCB1or ABCG2 mediated resistance to paclitaxel, doxorubicin and vincristine in HEK293/ABCB1 cells or HEK293/ABCG2 cells rather than the corresponding parental cell lines, and the reversal effects were also concentration dependen**ce** (Table 1). The results suggested that FW-04-806 potentiated the sensitivity of ABCB1 and ABCG2 overexpressing cells to substrate chemotherapeutic agents such as DOX, VCR, paclitaxel, mitoxantrone and topotecan in vitro. Taken together, FW-04-806 was able to reverse MDR of ABCB1 or ABCG2 overexpressing cells to substrate chemotherapeutic agents, instead of non-substrate agents.

**Table 1:** MDR reversal effect of FW-04-806 on ABCBl or ABCG2 substrate drugs

Compound	IC_50_ ± SD μM (fold-reversal)				Fold-resistance
	KB		KBv200(ABCB1)		
Doxorubicin	0.0346 ± 0.0061	(1.00)	2.9903 ± 0.3484	(1.00)	86.4
+ 1.25 μM FW-04-806	0.0360 ± 0.0086	(0.96)	1.1805 ± 0.3313^**^	(2.53)	
+ 2.5 μM FW-04-806	0.0312 ± 0.0045	(1.11)	0.5546 ± 0.2563^**^	(5.39)	
+ 5 μM FW-04-806	0.0341 ± 0.0197	(1.01)	0.4107 ± 0.0603^**^	(7.28)	
+ 10 μM Verapamil	0.0406 ± 0.01140	(0.85)	0.4147 ± 0.1578^**^	(7.21)	
Vincristine	0.0039 ± 0.0001	(1.00)	4.4343 ± 0.8370	(1.00)	1335.9
+ 1.25 μM FW-04-806	0.0046 ± 0.0000	(0.84)	0.1236 ± 0.0325^**^	(35.87)	
+ 2.5 μM FW-04-806	0.0039 ± 0.0000	(0.98)	0.0518 ± 0.0113^**^	(85.64)	
+ 5 μM FW-04-806	0.0030 ± 0.0000	(1.30)	0.0156 ± 0.0034^**^	(284.86)	
+ 10 μM Verapamil	0.0030 ± 0.0002	(1.31)	0.0637 ± 0.0122^**^	(69.62)	
Paclitaxel	0.0023 ± 0.0018	(1.00)	3.9227 ± 2.3426	(1.00)	1705.5
+ 1.25 μM FW-04-806	0.0026 ± 0.0006	(0.90)	0.1044 ± 0.0474^*^	(37.59)	
+ 2.5 μM FW-04-806	0.0028 ± 0.0006	(0.81)	0.0457 ± 0.0042^*^	(85.80)	
+ 5 μM FW-04-806	0.0017 ± 0.0001	(1.38)	0.0127 ± 0.0038^*^	(308.55)	
+ 10 μM Verapamil	0.0026 ± 0.0003	(0.88)	0.0688 ± 0.0394^*^	(57.06)	
	K562		K562/adr (ABCB1)		
Doxorubicin	0.0382 ± 0.0002	(1.00)	5.1410 ± 1.8822	(1.00)	134.6
+ 1.25 μM FW-04-806	0.0283 ± 0.0006	(1.35)	5.8430 ± 1.1266	(0.88)	
+ 2.5 μM FW-04-806	0.0307 ± 0.0011	(1.25)	2.0657 ± 0.9445	(2.49)	
+ 5 μM FW-04-806	0.0372 ± 0.0027	(1.03)	0.5321 ± 0.0667^*^	(9.66)	
+ 10 μM Verapamil	0.0402 ± 0.0019	(0.95)	0.7843 ± 0.1870^*^	(6.55)	
Vincristine	0.0007 ± 0.0000	(1.00)	0.1708 ± 0.0051	(1.00)	244.0
+ 1.25 μM FW-04-806	0.0007 ± 0.0000	(1.03)	0.0368 ± 0.0027^**^	(4.65)	
+ 2.5 μM FW-04-806	0.0006 ± 0.0000	(1.19)	0.0193 ± 0.0012^**^	(8.84)	
+ 5 μM FW-04-806	0.0007 ± 0.0000	(1.09)	0.0040 ± 0.0001^**^	(43.04)	
+ 10 μM Verapamil	0.0006 ± 0.0000	(1.18)	0.0027 ± 0.0002^**^	(64.21)	
Paclitaxel	0.0068 ± 0.0002	(1.00)	0.6002 ± 0.0175	(1.00)	88.3
+ 1.25 μM FW-04-806	0.0072 ± 0.0001	(0.94)	0.1401 ± 0.0014^**^	(4.28)	
+ 2.5 μM FW-04-806	0.0066 ± 0.0002	(1.02)	0.0707 ± 0.0011^**^	(8.49)	
+ 5 μM FW-04-806	0.0060 ± 0.0001	(1.12)	0.0253 ± 0.0013^**^	(23.76)	
+ 10 μM Verapamil	0.0072 ± 0.0001	(0.93)	0.0335 ± 0.0004^**^	(17.92)	
	MCF-7		MCF-7/adr (ABCB1)		
Doxorubicin	0.1653 ± 0.0066	(1.00)	8.9867 ± 0.8227	(1.00)	54.4
+ 1.25 μM FW-04-806	0.2010 ± 0.0397	(0.82)	2.0513 ± 0.1232^**^	(4.38)	
+ 2.5 μM FW-04-806	0.1870 ± 0.0608	(0.88)	0.8483 ± 0.0740^**^	(10.59)	
+ 5 μM FW-04-806	0.2215 ± 0.0486	(0.75)	0.4863 ± 0.0086^**^	(18.48)	
+ 10 μM Verapamil	0.1769 ± 0.0051	(0.93)	0.3688 ± 0.0460^**^	(24.37)	
	HEK293/pcDNA3.1		HEK293/ABCB1		
Doxorubicin	0.0836 ± 0.0001	(1.00)	1.7513 ± 0.0875	(1.00)	20.9
+ 2.5 μM FW-04-806	0.0794 ± 0.0017	(1.05)	0.2881 ± 0.0077^**^	(6.08)	
+ 5 μM FW-04-806	0.0726 ± 0.0005	(1.15)	0.1479 ± 0.0036^**^	(11.84)	
+ 10 μM FW-04-806	0.0879 ± 0.0004	(0.95)	0.1467 ± 0.0055^**^	(11.94)	
+ 10 μM Verapamil	0.0681 ± 0.0005	(1.23)	0.1868 ± 0.0002^**^	(9.37)	
	S1		S1-MI-80(ABCG2)		
Mitoxantrone	0.0409 ± 0.0007	(1.00)	48.7067 ± 5.6101	(1.00)	1190.9
+ 2.5 μM FW-04-806	0.0426 ± 0.0008	(0.96)	23.2267 ± 1.9806^**^	(2.10)	
+ 5 μM FW-04-806	0.0446 ± 0.0019	(0.92)	20.3067 ± 2.7537^**^	(2.40)	
+ 10 μM FW-04-806	0.0410 ± 0.0017	(1.00)	17.2100 ± 0.4694^**^	(2.83)	
+ 2.5 μM KO134	0.0483 ± 0.0075	(0.85)	0.3390 ± 0.0134^**^	(143.66)	
Topotecan	0.6221 ± 0.0639	(1.00)	168.1667 ± 18.0736	(1.00)	270.3
+ 2.5 μM FW-04-806	0.5729 ± 0.1353	(1.09)	138.7333 ± 3.2716	(1.21)	
+ 5 μM FW-04-806	0.4887 ± 0.0452	(1.27)	116.6667 ± 4.5170^**^	(1.44)	
+ 10 μM FW-04-806	0.5137 ± 0.0071	(1.21)	59.9400 ± 7.3335^**^	(2.81)	
+ 2.5 μM KO134	0.6414 ± 0.0178	(0.97)	11.7120 ± 2.9969^**^	(14.36)	
	H460		H460/MX20(ABCG2)		
Mitoxantrone	0.0110 ± 0.0001	(1.00)	1.0716 ± 0.0644	(1.00)	97.4
+ 2.5 μM FW-04-806	0.0109 ± 0.0003	(1.01)	0.3994 ± 0.0127^**^	(2.68)	
+ 5 μM FW-04-806	0.0115 ± 0.0005	(0.96)	0.2630 ± 0.0097^**^	(4.08)	
+ 10 μM FW-04-806	0.0109 ± 0.0002	(1.01)	0.2254 ± 0.0057^**^	(4.75)	
+ 2.5 μM KO134	0.0114 ± 0.0006	(0.97)	0.1728 ± 0.0015^**^	(6.20)	
Topotecan	0.0583 ± 0.0001	(1.00)	100.5367 ± 9.0013	(1.00)	1724.5
+ 2.5 μM FW-04-806	0.0591 ± 0.0015	(0.99)	66.9167 ± 1.1787^**^	(1.50)	
+ 5 μM FW-04-806	0.0582 ± 0.0002	(1.00)	53.4433 ± 7.4395^**^	(1.88)	
+ 10 μM FW-04-806	0.0546 ± 0.0004	(1.07)	38.3900 ± 3.9674^**^	(2.62)	
+ 2.5 μM KO134	0.0568 ± 0.0003	(1.02)	21.0567 ± 1.3238^**^	(4.77)	
	HEK293/pcDNA3.1		HEK293/ABCG2-R2		
Mitoxantrone	0.0054 ± 0.0006	(1.00)	0.4322 ± 0.0139	(1.00)	80.0
+ 1.25 μM FW-04-806	0.0035 ± 0.0000	(1.55)	0.2084 ± 0.0275^**^	(2.07)	
+ 2.5 μM FW-04-806	0.0059 ± 0.0001	(0.91)	0.1960 ± 0.0102^**^	(2.20)	
+ 5 μM FW-04-806	0.0034 ± 0.0001	(1.59)	0.1702 ± 0.0291^**^	(2.54)	
+ 2.5 μM KO134	0.0048 ± 0.0001	(1.11)	0.2990 ± 0.0009^**^	(1.45)	

Note: MTT assay was performed to analyze the cell survival after treatment with anticancer agents in the absence or presence of FW-04-806; VRP (specific inhibitor of ABCB1) and KO134 (a specific inhibitor of ABCG2) were used as the positive control. The fold reversal of MDR (values given in parentheses) was calculated by dividing the IC_50_ value for cells with the anticancer drug in the absence of FW-04-806 by that obtained in the presence of FW-04-806. Data was shown as mean ± SD from three independent experiments. ^*^, *P* < 0.05; ^**^, *P* < 0.01

**Table 2:** MDR reversal effect of FW-04-806 on ABCBl or ABCG2 non-substrate drugs

Compound	IC_50_ ± SD μM (fold-reversal)				Fold-resistance
	KB		KBv200(ABCB1)		
Cisplatin	3.8680 ± 1.3483	(1.00)	5.4893 ± 1.5035	(1.00)	1.4
+ 1.25 μM FW-04-806	2.0117 ± 0.1632	(1.92)	5.5650 ± 1.2172	(0.99)	
+ 2.5 μM FW-04-806	3.7403 ± 0.5553	(1.03)	4.0650 ± 0.4293	(1.35)	
+ 5 μM FW-04-806	2.2227 ± 0.3665	(1.74)	7.6887 ± 3.0405	(0.71)	
+ 10 μM Verapamil	1.8723 ± 0.3514	(2.07)	5.4337 ± 2.1716	(1.01)	
	K562		K562/adr (ABCB1)		
Cisplatin	1.0181 ± 0.2686	(1.00)	5.8687 ± 0.7091	(1.00)	5.8
+ 1.25 μM FW-04-806	1.0200 ± 0.0362	(1.00)	7.1563 ± 1.4092	(0.82)	
+ 2.5 μM FW-04-806	1.0510 ± 0.1333	(0.97)	7.7360 ± 0.5774	(0.76)	
+ 5 μM FW-04-806	1.1273 ± 0.0605	(0.90)	6.4127 ± 0.9883	(0.92)	
+ 10 μM Verapamil	0.8974 ± 0.0791	(1.13)	6.0500 ± 1.6236	(0.97)	
	MCF-7		MCF-7/adr (ABCB1)		
Cisplatin	15.0700 ± 0.3928	(1.00)	18.0167 ± 0.4153	(1.00)	1.2
+ 1.25 μM FW-04-806	18.7367 ± 0.1401	(0.80)	18.0933 ± 1.0193	(1.00)	
+ 2.5 μM FW-04-806	13.7733 ± 0.5398	(1.09)	18.9100 ± 0.2905	(0.95)	
+ 5 μM FW-04-806	16.4433 ± 0.4456	(0.92)	16.7633 ± 0.0981	(1.07)	
+ 10 μM Verapamil	18.4767 ± 1.1412	(0.82)	20.6867 ± 0.9530	(0.87)	
	HEK293/pcDNA3.1		HEK293/ABCB1		
Cisplatin	6.8007 ± 0.0765	(1.00)	8.6940 ± 0.1404	(1.00)	1.3
+ 2.5 μM FW-04-806	5.7683 ± 0.0093	(1.18)	9.2183 ± 0.2919	(0.94)	
+ 5 μM FW-04-806	5.6213 ± 0.0837	(1.21)	7.5417 ± 0.2921	(1.15)	
+ 10 μM FW-04-806	5.1467 ± 0.0979	(1.32)	7.7477 ± 0.0916	(1.12)	
+ 10 μM Verapamil	5.2640 ± 0.0900	(1.29)	7.3300 ± 0.2154	(1.19)	
	S1		S1-MI-80(ABCG2)		
Cisplatin	12.7233 ± 0.5525	(1.00)	12.8800 ± 0.0608	(1.00)	1.01
+ 2.5 μM FW-04-806	12.9067 ± 0.4382	(0.99)	13.3100 ± 0.6678	(0.97)	
+ 5 μM FW-04-806	15.1167 ± 0.3386	(0.84)	17.8867 ± 0.3785	(0.72)	
+ 10 μM FW-04-806	13.2400 ± 0.1967	(0.96)	14.6533 ± 0.4388	(0.88)	
+ 2.5 μM KO134	15.5033 ± 0.1124	(0.82)	16.2633 ± 0.2538	(0.79)	
	H460		H460/MX20(ABCG2)		
Cisplatin	4.1593 ± 0.0618	(1.00)	15.9633 ± 0.3653	(1.00)	3.8
+ 2.5 μM FW-04-806	3.4410 ± 0.3634	(1.21)	15.8067 ± 0.5622	(1.01)	
+ 5 μM FW-04-806	3.2287 ± 0.0302	(1.29)	17.2300 ± 0.2685	(0.93)	
+ 10 μM FW-04-806	3.8967 ± 0.0093	(1.07)	14.8167 ± 0.2974	(1.08)	
+ 2.5 μM KO134	4.0677 ± 0.1045	(1.02)	17.0767 ± 1.3276	(0.93)	
	HEK293/pcDNA3.1		HEK293/ABCG2-R2		
Cisplatin	6.8007 ± 0.0765	(1.00)	3.8733 ± 0.1240	(1.00)	0.57
+ 1.25 μM FW-04-806	6.1203 ± 0.0740	(1.11)	3.5323 ± 0.1928	(1.10)	
+ 2.5 μM FW-04-806	6.0470 ± 0.0252	(1.12)	4.6223 ± 0.1293	(0.84)	
+ 5 μM FW-04-806	6.4633 ± 0.2812	(1.05)	3.9990 ± 0.0717	(0.97)	
+ 2.5 μM KO134	6.8980 ± 0.2655	(0.99)	4.7723 ± 0.4980	(0.81)	

Note: MTT assay was performed to analyze the cell survival after treatment with anticancer agents in the absence or presence of FW-04-806; VRP (specific inhibitor of ABCB1) and KO134 (specific inhibitor of ABCG2) were used as the positive control. The fold reversal of MDR (values given in parentheses) was calculated by dividing the IC_50_ value for cells with the anticancer drug in the absence of FW-04-806 by that obtained in the presence of FW-04-806. Data was shown as mean ± SD from three independent experiments

### FW-04-806 enhanced the anticancer effect of vincristine in ABCB1 overexpressing KBv200 cell xenografts model in vivo

**Fig. 2:**
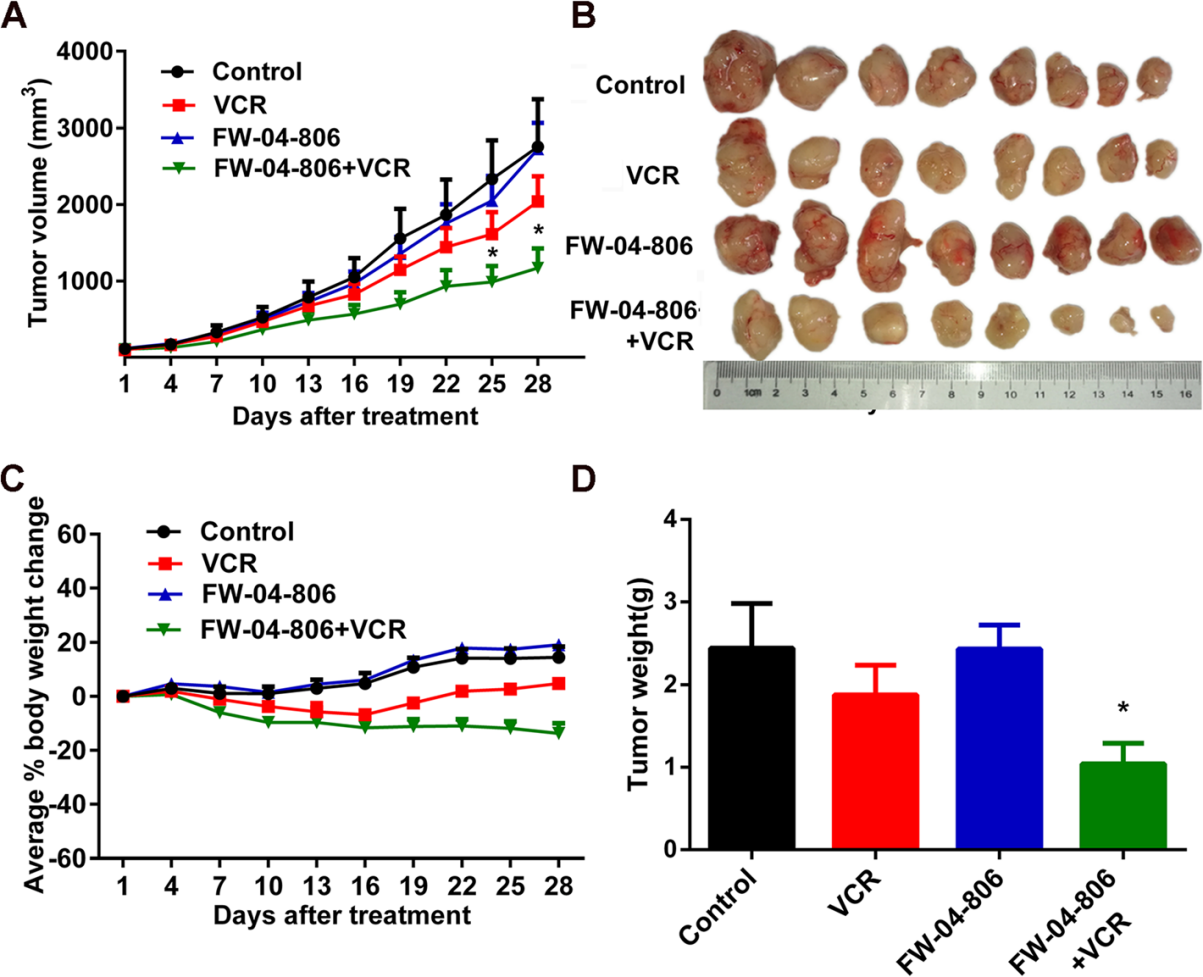
The potentiation of the anticancer effect of vincristine by FW-04-806 in the KBv200 cell xenograft nude mice model. **a** The tumor growth curve was drawn according to the tumor volume and day after treatment. Data shown are mean ± SD of tumor volume for each group (*n* = 8). **b** The image of tumors size in four groups excised from the mice on the 28th day after implantation. **c** Animals’ body weights were measured every 3 days, and the average percentage change was calculated. **d** The average tumor weight of each group was calculated after the tumors excised from the mice. The four treatment groups were: (1) saline (i.g., every 3 day); (2) vincristine (0.5 mg/kg, i.v., every 3 day); (3) FW-04-806 (100 mg/kg, i.g., every 3 day); (4) FW-04-806 (100 mg/kg, i.g., every 3 day, given 1 h before vincristine administration) plus vincristine (0.5 mg/kg, i.v., every 3 day). Data shown are mean ± SD for each group; ^*^ *P* < 0.05

To investigate whether FW-04-806 could reverse ABCB1-mediated MDR in vivo, we established the ABCB1 overexpressing multidrug-resistant KBv200 cell xenograft model in nude mice. Corresponding to the use of non-cytotoxic concentration as the reversal dose in vitro, the dose with no significant inhibition on tumor growth was selected as the reversal dose of MDR in vivo, which was determined to be 100 mg/kg by preliminary experiments. There was no significant difference in tumor size between animals treated with vehicle, FW-04-806 or vincristine alone (Fig. 2a). However, the group of combination of FW-04-806 with vincristine had greater inhibitory effect on tumor growth compared with vincristine or FW-04-806 alone group (*P* < 0.05; Fig. 2a, b, and d), and the inhibition rate of tumor weight was 57.39%. Furthermore, no significant weight loss or mortality was observed in the mice. These data suggested that FW-04-806 had significant reversal effect on ABCB1-mediated MDR in vivo without observed adverse effect.

### FW-04-806 increased the intracellular accumulation of DOX or Rho 123 in ABCB1 or ABCG2 overexpressing cells

**Fig. 3:**
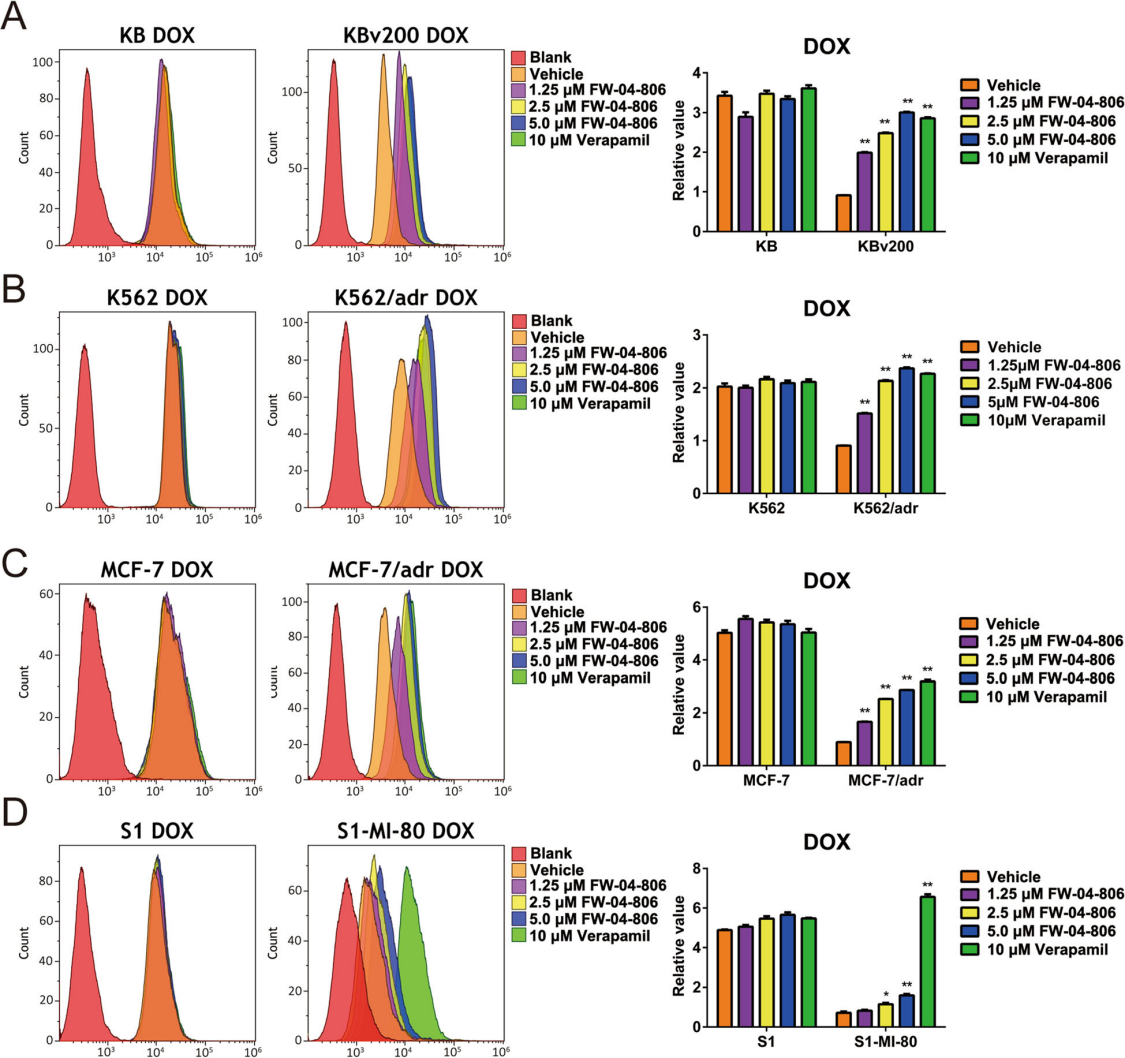
Effect of FW-04-806 on the intracellular accumulation of DOX in MDR cells and their parental sensitive cells. The intracellular accumulation of DOX in KBv200 and KB (**a**), K562/adr and K562 (**b**), MCF-7/adr and MCF-7 (**c**), S1-MI-80 and S1 cells (**d**) was measured by flow cytometric analysis. A representative diagram from three independent experiments was shown. The accumulation of DOX was quantified as fold change in fluorescence intensity of DOX relative to the control MDR cells treated with vehicle and DOX, it was calculated with dividing the fluorescence intensity of each sample by that of MDR cells treated with vehicle and DOX. Data are expressed as mean ± SD from three independent experiments. ^*^ *P* < 0.05; ^**^ *P* < 0.01

**Fig. 4:**
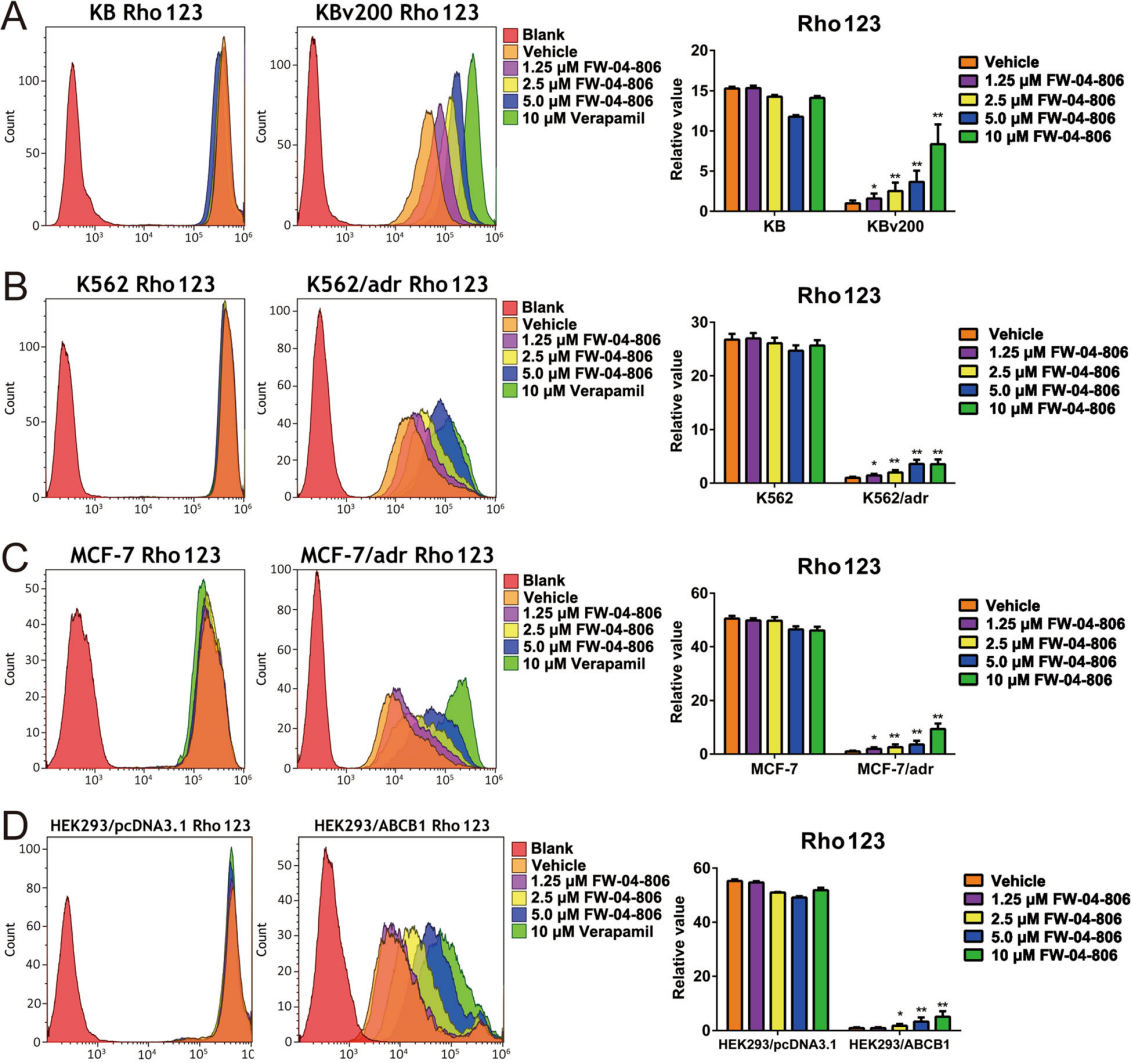
Effect of FW-04-806 on the intracellular accumulation of Rho123 in MDR cells and their parental sensitive cells. The intracellular accumulation of Rho123 in KBv200 and KB (**a**), K562/adr and K562 (**b**), MCF-7/adr and MCF-7 (**c**), HEK293/ABCB1 and HEK293/pcDNA3.1 cells (**d**) was measured by flow cytometric analysis. A representative result from three independent experiments was shown. The accumulation of Rho123 was quantified as fold change in fluorescence intensity of Rho123 relative to the control MDR cells treated with vehicle and Rho123, it was calculated with dividing the fluorescence intensity of each sample by that of MDR cells treated with vehicle and Rho123. Data are expressed as mean ± SD from three independent experiments. ^*^ *P* < 0.05; ^**^ *P* < 0.01

The significantly synergistic effect from combination of FW-04-806 with substrate anticancer drugs in vitro and in vivo suggested its reversal MDR effects. Therefore, we detected the effect of FW-04-806 on the intracellular accumulation of DOX or Rho 123, the substrate agents of ABC transporters, in ABCB1 or ABCG2 overexpressing cell lines (KBv200, K562/adr, MCF-7/adr, HEK293/ABCB1 and S1-MI-80) by flow cytometry analysis. The intracellular accumulation of DOX or Rho 123 was significantly lower in ABCB1 or ABCG2 overexpressing resistant cells than that in their parental sensitive cells. When treated with FW-04-806, the intracellular accumulations of DOX (Fig. 3) or Rho 123 (Fig. 4) significantly increased in the resistant cells, rather than in their parental sensitive cells. The present findings suggested that FW-04-806 was able to inhibit the function of ABCB1 and ABCG2, subsequently increased the intracellular accumulation of DOX and Rho 123 in ABCB1 or ABCG2 overexpressing cells.

### FW-04-806 inhibited the efflux of DOX in ABCB1 or ABCG2 overexpressing cells

**Fig. 5:**
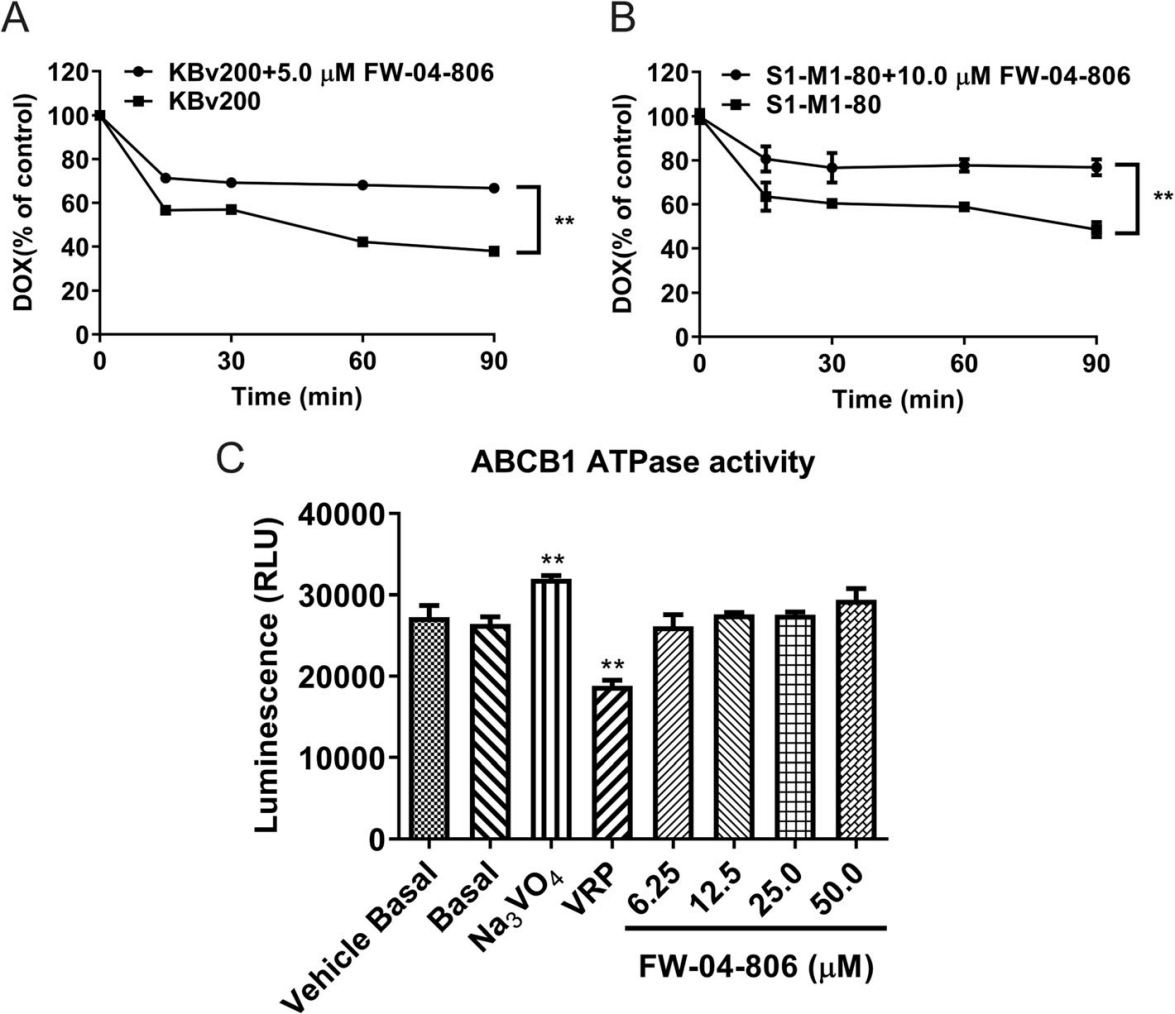
Effect of FW-04-806 on the efflux of DOX and the ATPase activity of ABCB1. The DOX retention was measured by flow cytometry in KBv200 (**a**) and S1-MI-80 cells (**b**). The cells were preincubated with 10 μmol/L of DOX at 37 °C for 3 h and then incubated with or without FW-04-806 for indicated time points in DOX-free media, the samples at different time point were measured by flow cytometry. Effect of FW-04-806 on ATPase activity of ABCB1 (**c**). Data represent mean ± SD from three independent experiments. ^**^P < 0.01

To determine whether the increased intracellular accumulation of DOX was due to the inhibitory effect of FW-04-806 on MDR cell efflux, we performed an efflux assay. The efflux of DOX over 90 min after an initial drug accumulation was monitored. As expected, after 90 min efflux DOX retention dropped remarkably from 100% (0 min) to 37.9% (90 min) in ABCB1 overexpressing KBv200 cells (Fig. 5a), or from 100% (0 min) to 48.5% (90 min) in ABCG2 overexpressing S1-MI-80 cells (Fig. 5b). While after pretreatment with FW-04-806 5 μM in KBv200 cells or 10 μM in S1-MI-80 cells, the efflux of DOX was significantly inhibited, therefore it resulted in an increase of DOX retention in KBv200 cells from 37.9 to 66.8% (Fig. 5a) or in S1-MI-80 cells from 48.5 to 76.8% (Fig. 5b) at the 90 min time point. The results showed that FW-04-806 inhibited efflux function of ABC transporter for substrate drugs in ABCB1 or BCG2 overexpressing cells.

### FW-04-806 have no significant effect on the ATPase activity of ABCB1

A crucial role of ABC transporters in the development of MDR is achieved by pumping out drugs from the cells, and this process is coupled to the energy of ATP hydrolysis on the ATPase domain of ABC transporters, and most transport substrates were able to stimulate the activity of the ATPase. We used ABCB1 ATPase Activity Assay Kit (Promega, V3601) to evaluated the ABCB1 ATPase activity in the presence of indicated concentrations of FW-04-806, VRP and Na_3_VO_4_. The assay relies on the ATP-dependence of the light-generating reaction of luciferase. After a pool of ATP is first exposed to the ATPase, ATP consumption is detected as a decrease in luminescence from a second reaction with a recombinant luciferase, thus the greater the decrease in signal, the higher the ATPase activity. The results showed that VRP could increase the ATPase activity of ABCB1, Na_3_VO_4_ inhibited that, while FW-04-806 had no significant effect on the ATPase activity of ABCB1 (Fig. 5c).

### FW-04-806 did not obviously alter the expression levels of ABCB1 and ABCG2

**Fig. 6:**
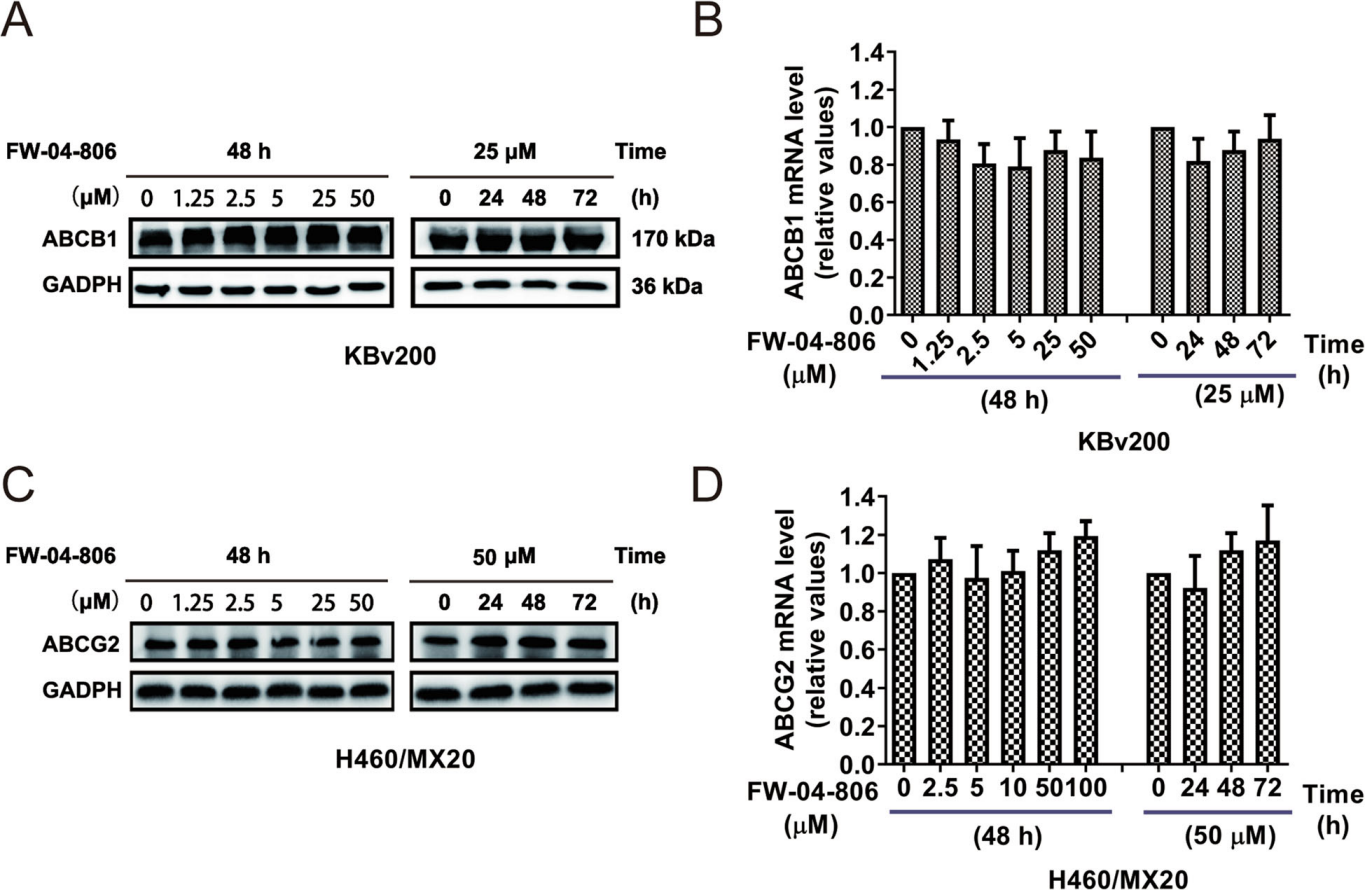
Effect of FW-04-806 on the expression of ABCB1, ABCG2. The protein level of ABCB1 (**a**) and ABCG2 (**c**) were detected by Western blot assay. Real-time quantitative PCR assay was performed to quantify the mRNA levels of ABCB1 in KBv200 (**b**) and ABCG2 in H460/MX20 cells (**d**). A representative result from three independent experiments was shown in each panel. Data represent mean ± SD from three independent experiments

The reversal of ABCB1 and ABCG2 mediated MDR could be achieved by either inhibiting ABC transporter function or downregulating their expression levels. Therefore, the effect of FW-04-806 on the expression of ABCB1 and ABCG2 was detected by Western blot and qRT-PCR assay (Fig. 6). The results showed that FW-04-806 did not obviously alter the protein or mRNA expression levels of both ABCB1 and ABCG2, even up to the concentration of 50 μmol/L (in KBv200 cells) and 100 μmol/L (in H460/MX20 cells) and the extension of exposure time to 72 h. The results revealed that reversal MDR of FW-04-806 was through inhibiting the transport function of ABCB1 ro ABCG2, but not by downregulating their expression.

### Molecular docking and binding site analysis

**Fig. 7:**
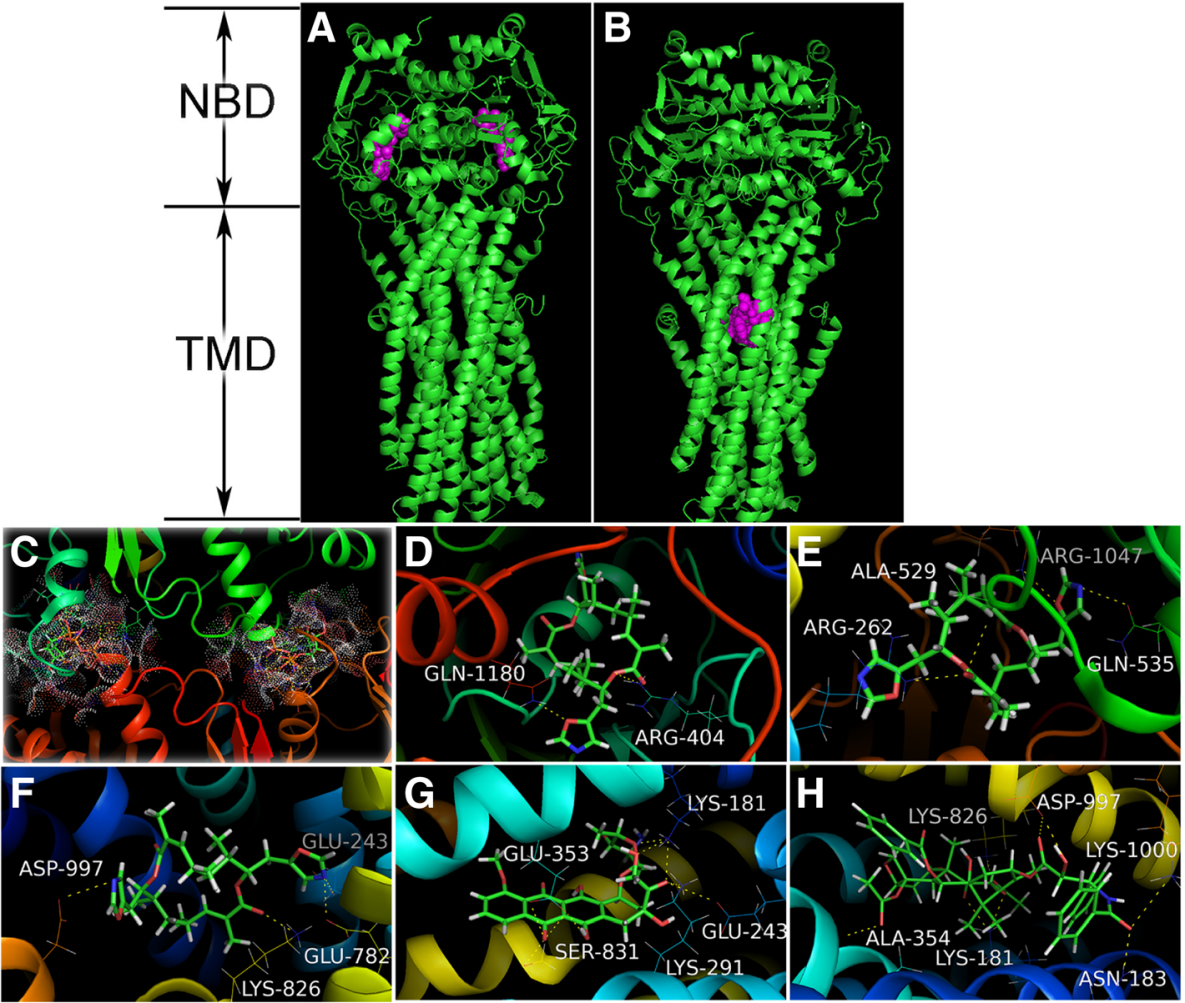
FW-04-806 docking to human P-glycoprotein (PDB ID: 6C0V). **a** Two symmetrical ATPase sites (purple) in NBDs. **b** The drug-substrate binding site (pink) in TMDs. **c** The binding mode of ATP at ATPase site 1(white dot on the right) and ATPase site 2 (white dot on the left). **d~e** Detailed view of FW-04-806 at ATPase site 1 (**d**) and ATPase site 2 (**e**). **f~h** Detailed view of FW-04-806 (**f**), DOX (**g**) and PTX (**h**) in the drug-substrate binding site and hydrogen bonds (yellow) of drugs with TMDs

The molecular structure of human P-glycoprotein (PDB ID: 6C0V) contains two transmembrane domains (TMDs) and two symmetrical ATPase sites in NBDs (Fig. 7a and b). Firstly, we docked ATP or FW-04-806 to the ATP-binding sites, which exhibited that ATP had high docking scores of 14.16 at the ATP-binding site 1 and 12.20 at the ATP-binding site 2 (Fig. 7c). However, FW-04-806 had a docking score of 4.09 at the ATP-binding site 1 and 1.48 at the ATP-binding site 2 (Fig. 7d and e). It suggested that the affinity of FW-04-806 to the ATPase sites was much lower than that of ATP, which was consistent with the result that showed no significant effect of FW-04-806 on the ATPase activity of ABCB1 (Fig. 5c). Then, FW-04-806 was docked to TMDs, the “best” protomol was generated inside the TMDs of ABCB1 (Fig. 7b) with a highest docking score of 7.47 (Fig. 7f). Thus, we chose this protomol to dock DOX or PTX to TMDs, the docking scores were 5.71 and 6.89 respectively (Fig. 7g and h). These results suggested that DOX, PTX and FW-04-806 were all well-fitted at the binding site. However compared to DOX or PTX, FW-04-806 had a higher docking score in the drug-substrate binding site in TMDs of ABCB1, This could be attributed to FW-04-806 forming four hydrogen bond with Glu243 in TM4, Glu782 in TM8, Lys826 in TM9 and Asp997 in TM12 within TMDs. And we led to infer that FW-04-806 bound to drug-substrate binding site in TMDs more stably than substrate anticancer drugs, therefore it might obstruct the substrate anticancer drugs binding to the site and inhibited them pumped out of the cells.

## Discussion

This work revealed that FW-04-806 (conglobatin) significantly enhanced the cytotoxicity of substrate chemotherapeutic agents on ABCB1 or ABCG2 overexpressing cells in vitro and in vivo which showed its reversal effect for MDR mediated by ABCB1 or ABCG2. The effect was due to FW-04-806 via effectively inhibiting substrate efflux to produce increase of intracellular substrate chemotherapeutic agents in MDR cell lines. However, FW-04-806 did not alter the mRNA or protein expression level of ABCB1 and ABCG2 in reversal MDR or even higher concentration. Taken together, FW-04-806 reversed MDR by inhibiting the efflux function of ABC transporters subsequently increasing the intracellular concentration of substrate chemotherapeutic agents with no effect on expression of the transporters.

However, what is the possible mechanism in which FW-04-806 inhibits the efflux function of the transporters? In the majority of ABC transporters, ATP hydrolysis is mediated by two nucleotide-binding domains (NBDs), which closely interacts with two transmembrane domains (TMDs). Conformational changes occurring at the level of NBDs, upon ATP hydrolysis are further transmitted to TMDs, which form the dynamic channel through which the substrates pumped out of the cell [35]. As substrate translocation by ABC transporters is fueled by the energy of ATP hydrolysis, and substrates in most cases increase the turnover and ATP hydrolysis of the transporter, therefore we tested the effect of FW-04-806 on the ATPase activity. But FW-04-806 exhibited no influence on ATPase activity of ABCB1, which is not like many identified reversal agents (e.g., Verapamil) that stimulated the basal ATPase activity. The pattern of their interactions can be inferred from the molecular docking of FW-04-806 and ABCB1. It was found that the affinity between FW-04-806 and the ATP binding site in NBDs of ABCB1 is much lower than that of ATP, which may explain why FW-04-806 has no significant influence on ATPase of ABCB1. The docking of FW-04-806 with the substrate binding site of TMD in ABCB1 suggests that FW-04-806 has a higher affinity to the substrate binding site than the substrate anticancer drugs. Therefore, it can be imagined that FW-04-806 may preferentially bind with the substrate binding site and reject substrate anticancer drugs binding to the site, which may inhibit the conformational changes of TMD and thus suppress the efflux of the substrate anticancer drugs, increase the intracellular concentration of substrate chemotherapeutic agents, and result in reversing MDR.

## Conclusions

In conclusion, we found for the first time that FW-04-806 (conglobatin), a macrocyclic dilactone compound obtained from the fermentation products of streptomyces, can significantly reverse MDR in ABCB1 and ABCG2 over expression cells at the non-cytotoxic concentration, and significantly enhance the antitumor effect of vincristine, a substrate anticancer drug, in ABCB1 overexpressing KBv200 cell xenografts in vivo. The mechanism of its reversal MDR may be related to the inhibition of the efflux pump function of ABC transporters and then increase of the intracellular concentration of substrate anticancer drugs. However, FW-04-806 has no influence on the expression of ABCB1 or ABCG2 in MDR cells both at protein and mRNA levels, nor on the ATPase activity of ABC transporters. FW-04-806 is a compound that has both anti-tumor and reversal MDR effect, and its antitumor clinical application is worth further study.

## Data Availability

All data generated or analyzed during this study are included either in this article or in the supplementary information files.

## Abbreviations

ABC transporter: ATP-binding cassette transporter
ABCB1: ATP binding cassette sub-family B member 1
ABCC1: ATP binding cassette sub-family C member 1
ABCG2: ATP binding cassette sub-family G member 2
DOX: Doxorubicin
Hsp90: Heat shock protein 90
MDR: Multidrug resistance
MX: Mitoxantrone
NBDs: Nucleotide binding domains
PTX: Paclitaxel
TMDs: Transmembrane domains
VCR: Vincristine
VLB: Vinblastine
VRP: Verapamil
